# Supplementary material for: Recruitment of the m6A/m6Am demethylase FTO to target RNAs by the telomeric zinc finger protein ZBTB48
Source: Genome Biol. 2024 Sep 19;25:246. doi: 10.1186/s13059-024-03392-7 (PMC11414060; doi:10.1186/s13059-024-03392-7)

Fig 1

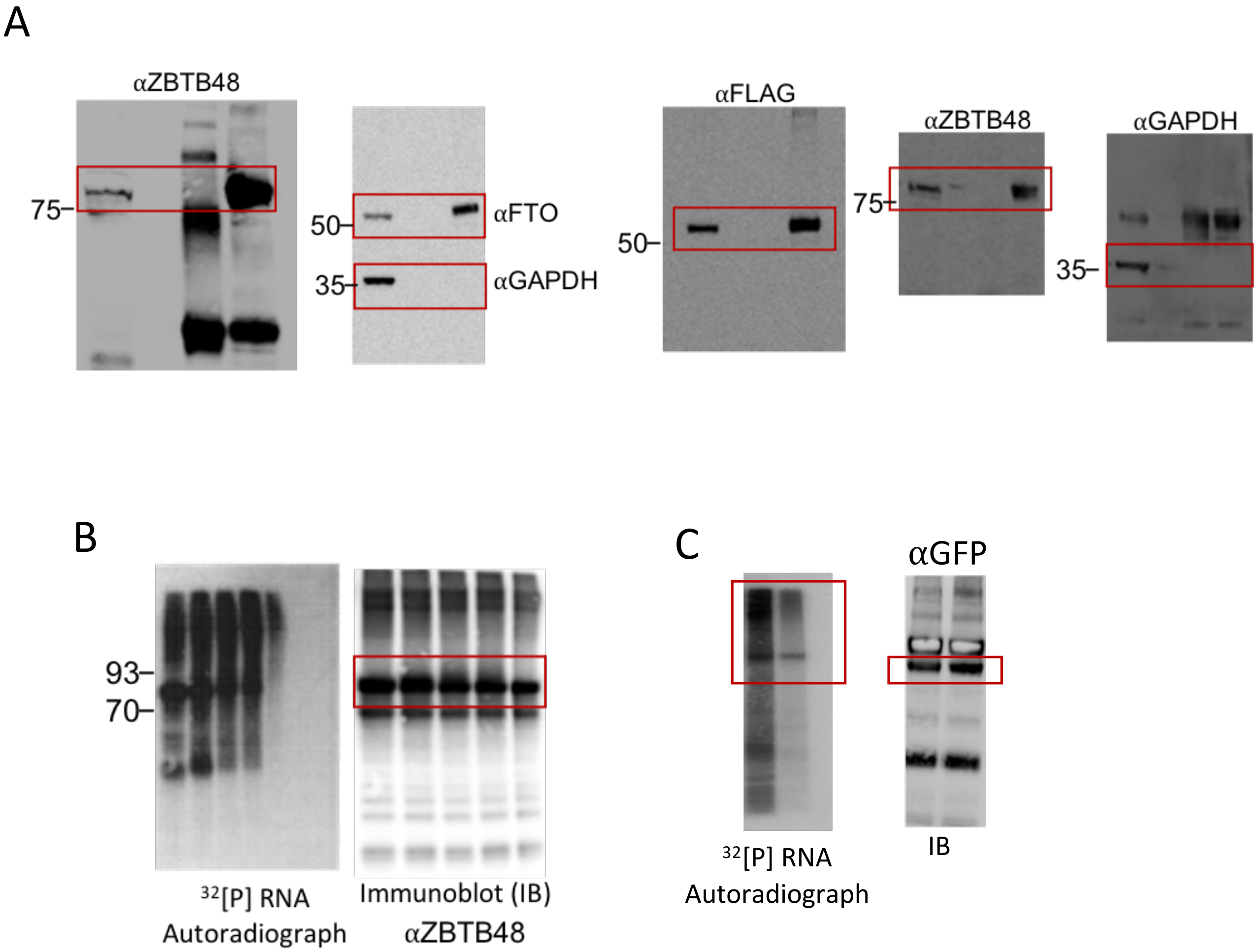
A
αZBTB48
75
50
35
αFTO
αGAPDH
αFLAG
50
αZBTB48
75
αGAPDH
35
B
93
70
32[P] RNA
Autoradiograph
Immunoblot (IB)
αZBTB48
C
αGFP
32[P] RNA
Autoradiograph
IB

Fig 2

H

J

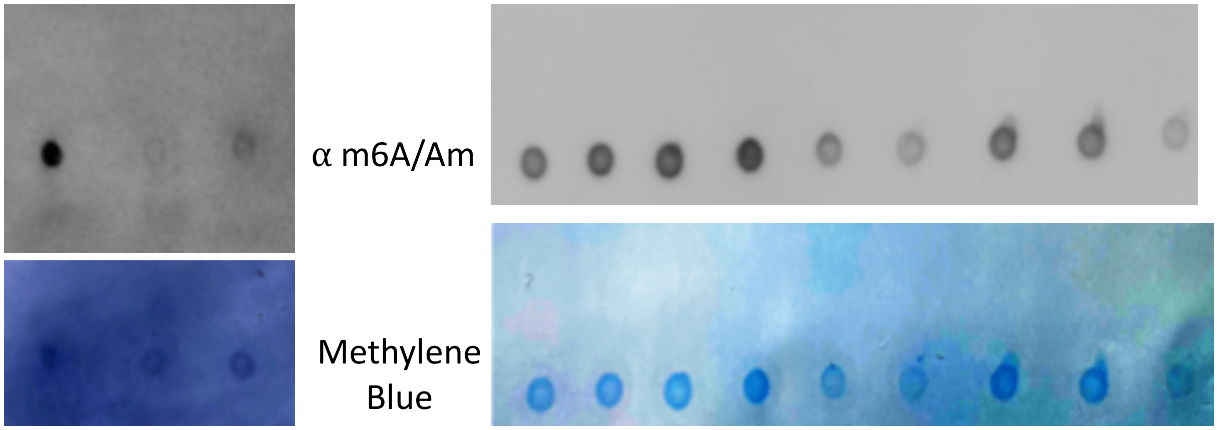

Figure 3A

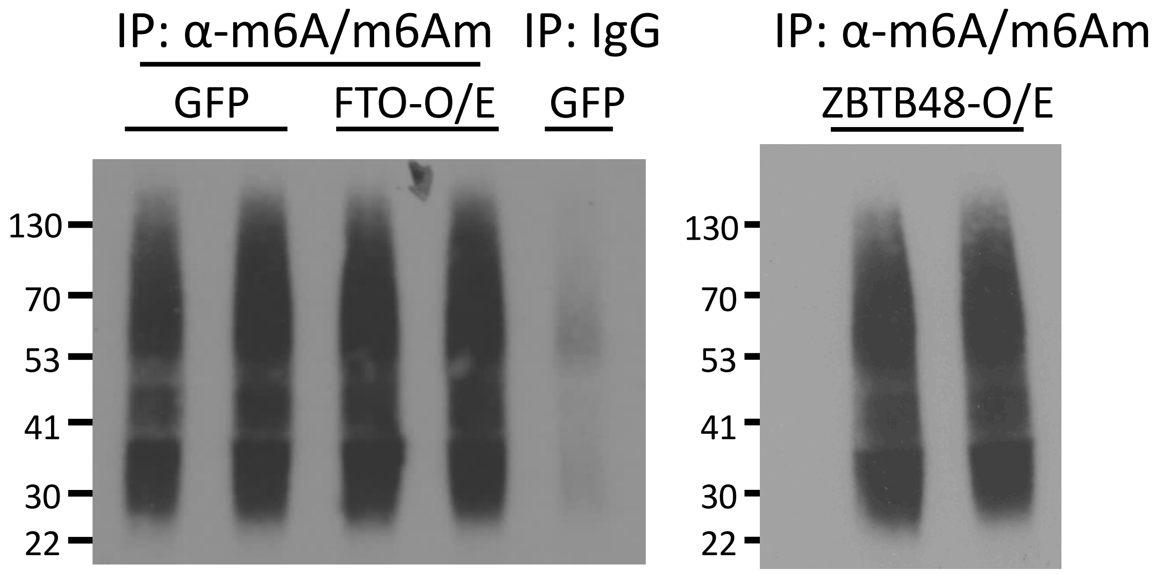

^{32}[P] RNA
Autoradiographs

Fig 5

D

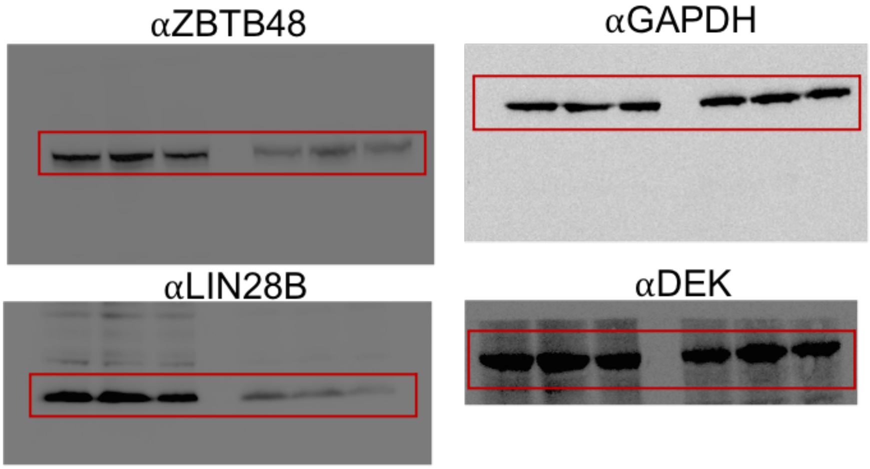

Membranes were cut based on protein sizes prior to antibody probing

H

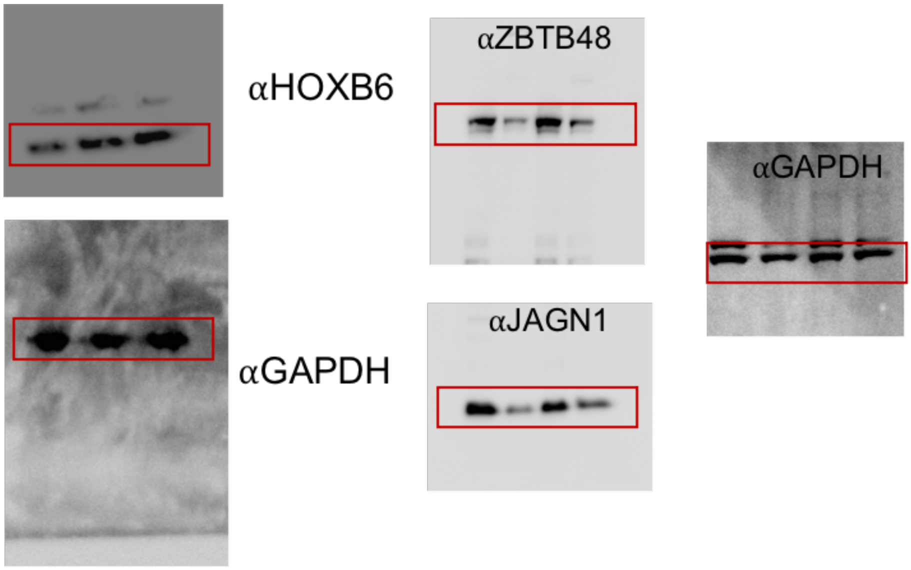

I

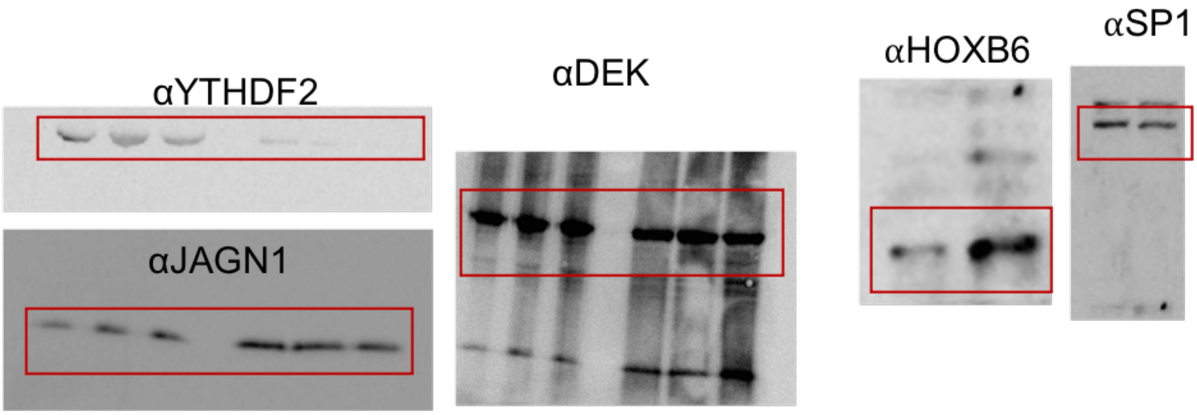

Fig 6G

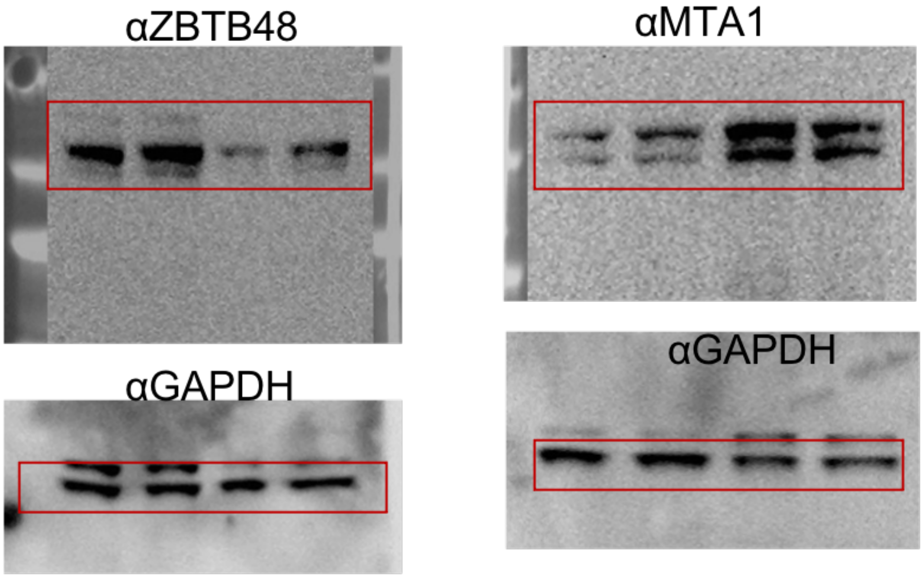

Fig S1

D

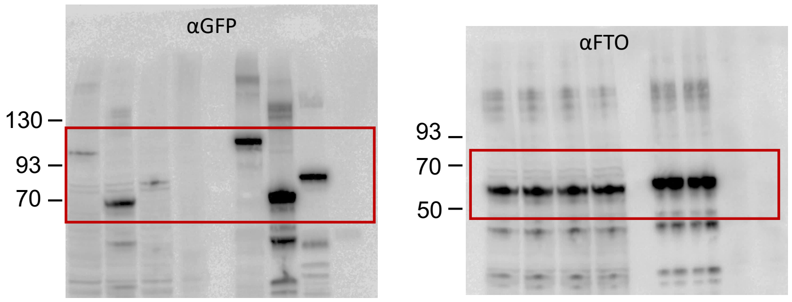

IP'd material was divided into two halves and resolved in parallel to probe for the indicated antibodies

E

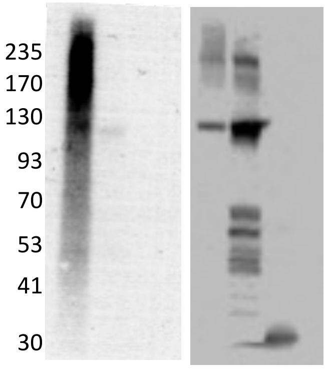

^{32}P Autoradiograph **αGFP immunoblot**

F

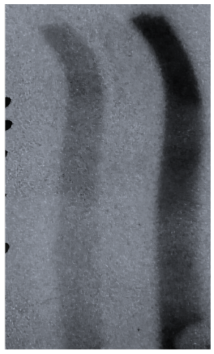

^{32}[P] RNA Autoradiograph

G

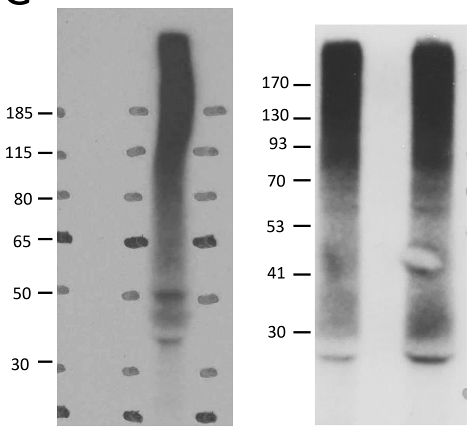

^{32}[P] RNA Autoradiograph ^{32}[P] RNA Autoradiograph

Fig S4

B

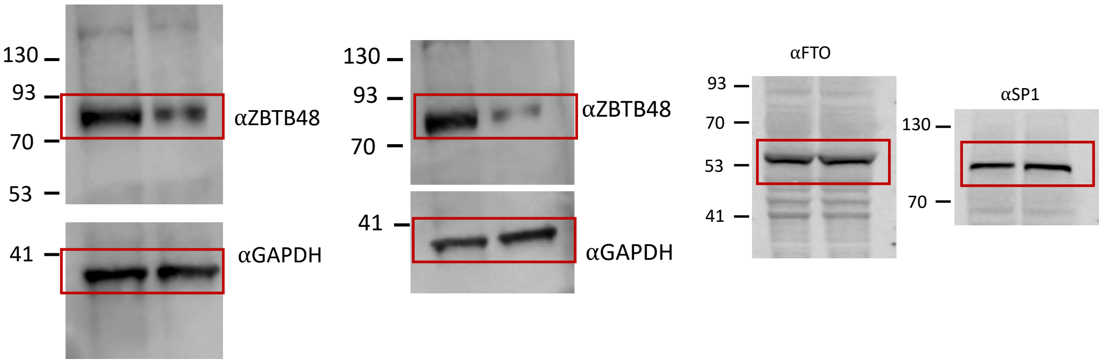

F

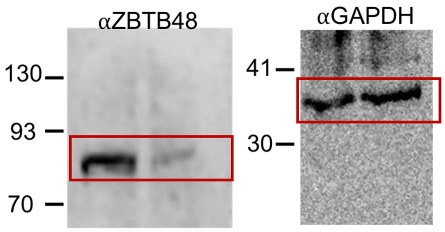

G

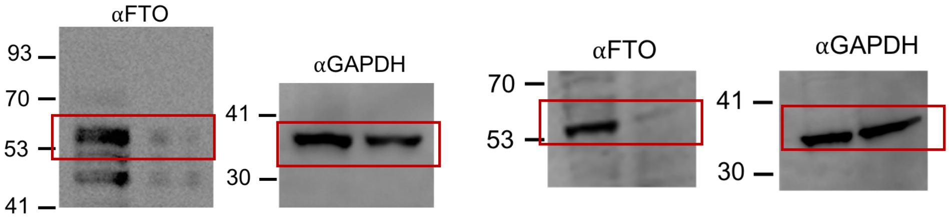

Figure S5G

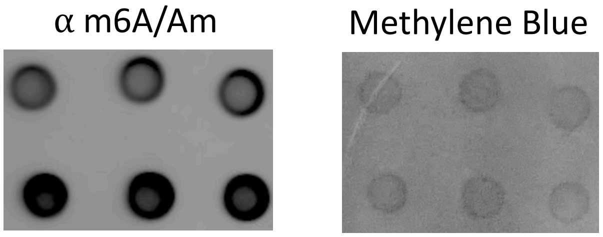

Fig S7C

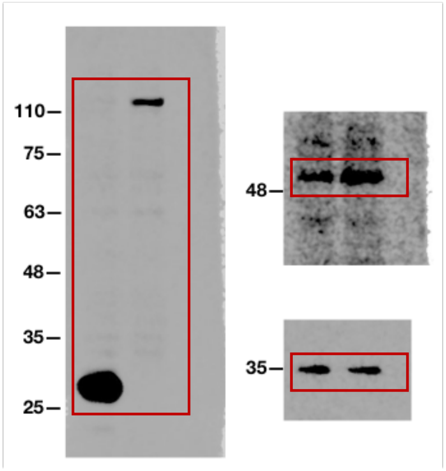

Figure S8

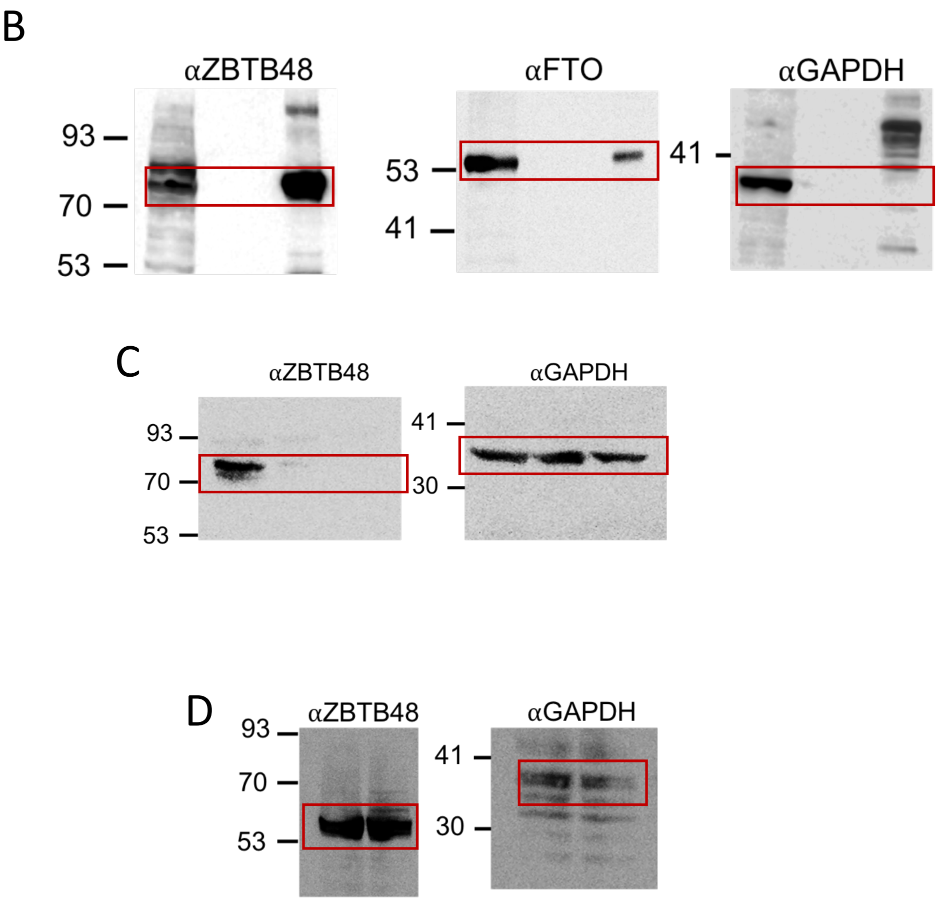

Fig S10

C

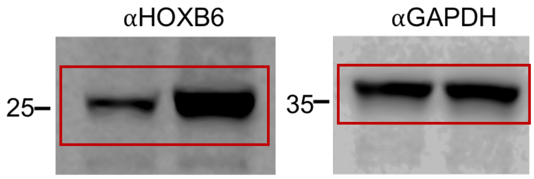
αHOXB6
αGAPDH
25
35

E

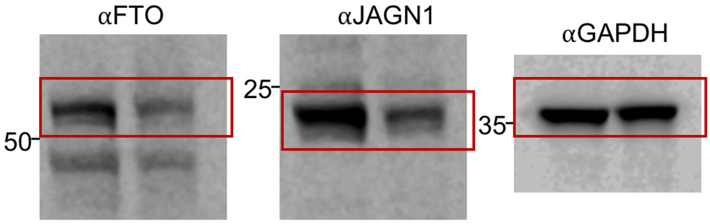
αFTO
αJAGN1
αGAPDH
50
25
35

F

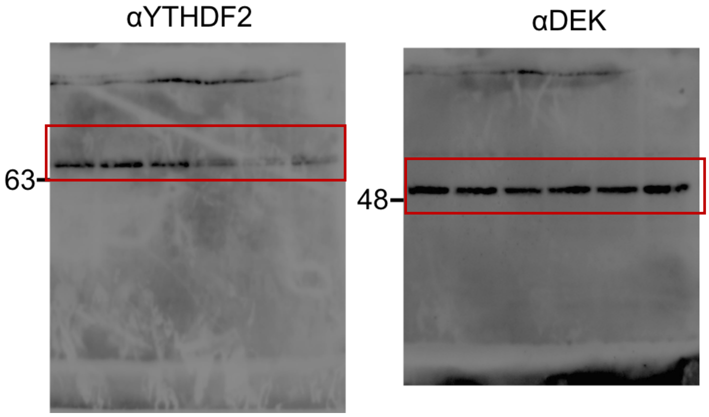
αYTHDF2
αDEK
63
48

Fig S11

A

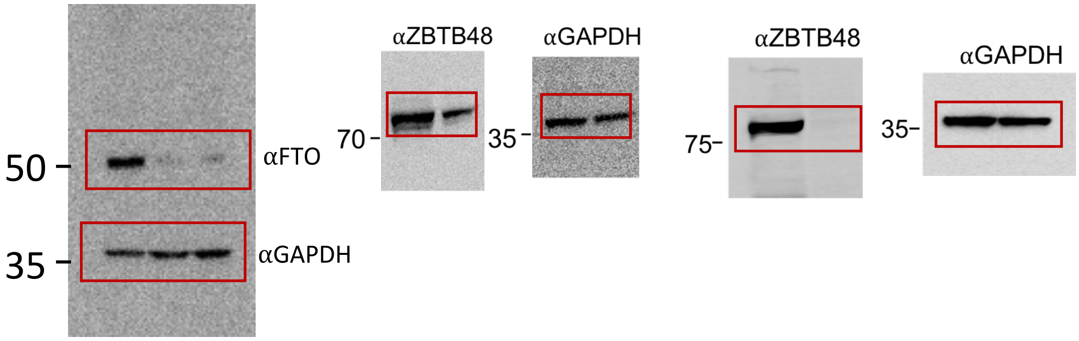

E

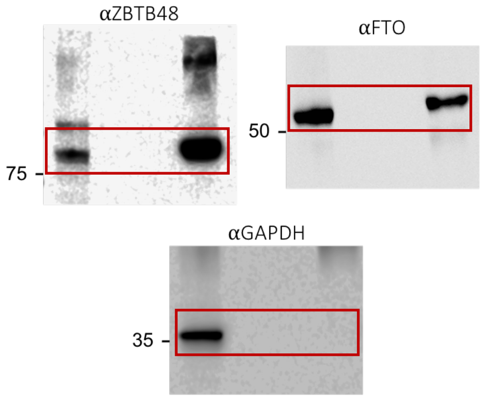

Figure S12D

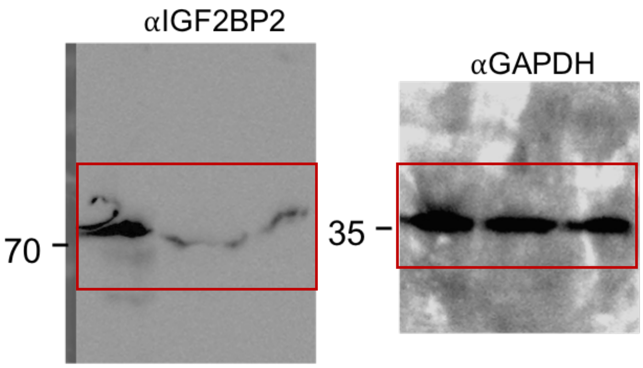

Supplement: Supplementary file 13 — Additional file 13. Uncropped Western blots. [file 13059_2024_3392_MOESM13_ESM.pdf]
