# Supplementary material for: Recruitment of the m6A/m6Am demethylase FTO to target RNAs by the telomeric zinc finger protein ZBTB48
Source: Genome Biol. 2024 Sep 19;25:246. doi: 10.1186/s13059-024-03392-7 (PMC11414060; doi:10.1186/s13059-024-03392-7)
Supplement: Supplementary file 14 — Additional file 14. Review history. [file 13059_2024_3392_MOESM14_ESM.docx]

Review History

**First round of review**

**Reviewer 1**

**Comments to author:**

Review Commons *Report 1*. **https://doi.org/10.15252/rc.2024693755.**

**Reviewer 2**

**Comments to author:**

Review Commons *Report 2*. [**https://doi.org/10.15252/rc.2024478417**](https://doi.org/10.15252/rc.2024478417).

**Authors Response**

**Point-by-point responses to the reviewers’ comments:**

Reviewer #1:

In this manuscript, Nabeel-Shah et al. identified that the telomeric zinc finger protein ZBTB48 help recruit FTO onto target RNA, including mRNAs and the telomere-associated regulatory RNA TERRA, to achieve cellular RNA m6A/m6Am demethylation, thereby regulating cell biology such as tumour growth studied in this study. The biochemistry and molecular biology experiments were done in HEK293 cells, and the cell models for tumour growth was done in HCT116 cells. However, I have identified both major and minor concerns that, when addressed, could further strengthen the findings and enhance the impact on the field.

*Authors’ response: We thank the reviewer for providing valuable feedback to improve our work. Below we provide our response to each point that the reviewer raised.*

Major concerns

(1) Regarding the ZBTB48/FTO targets and m6A/m6Am level that are regulated by ZBTB48/FTO axis.

Wei et al Molecular Cell 2018 (PMID: 30197295) (Figure 1B) quantified the m6A/m6Am levels upon knockdown of FTO in HeLa, HEK293, and 3T3-L1 cells, and found that the m6A/m6Am levels are generally mildly (10-20%) yet significantly upregulated on poly(A)+ RNAs. Here shown in the Figure 4A in this manuscript, the authors show that (1) siFTO and siZBTB48 led to ~2-3 fold upregulation of m6A and m6Am levels using total RNAs (dominant by rRNA in the population), and (2) that the m6A and m6Am levels are similar between siFTO and siZBTB48. Regarding (1), can the authors explain the discrepancy? This point is also relevant to the m6AIP-qRT-PCR results in this manuscript. Regarding (2), does this result suggest that ZBTB48 helps FTO to demethylate nearly all its targets, rather than a subset?

*Authors’ response: We agree with this reviewer that FTO depletion usually causes only a mild, although significant, increase in m6A/m6Am levels for polyA mRNAs, as reported previously. As pointed out by the reviewer, we used total RNA in Figure 4A, rather than polyA mRNAs (used in Wei et al), and this could account for the observed more significant global increase in the m6A levels upon FTO depletion. Moreover, experimental/technical differences (detailed procedures are provided in the Methods section) between the two studies may provide another source for the observed differences in FTO KD m6A ratios* *(relative to controls). In fact, several studies published subsequent to Wei et al, have shown ~2-3 fold upregulation in m6Am levels upon FTO depletion (e.g., PMID: 33741917, Nature Comm 2021, Fig 4C). Moreover, in multiple papers published by the same group (i.e., Chuan He’s lab), authors used m6A-IP qPCR and showed >2-3 fold upregulation in m6A levels in individual genes upon FTO knockdown (e.g., PMID: 33846348, Nature Comm 2021, Fig 3J, 3K; PMID: 33220174, Dev Cell 2020, Fig 6E; PMID: 31239444, Nature Comm 2019, Fig 4H). Thus, the accumulating evidence suggests that FTO depletion causes a substantial increase (although sometimes mild) in m6A/m6A levels, and consistently we also observed the same.*

*Importantly, consistent with Wei et al. 2019, we also found that FTO-overexpression caused only a mild decrease in global m6A/m6Am levels in polyA mRNAs (Figure S7F, G). It should be noted, however, that ZBTB48 overexpression results in a more pronounced decrease in m6A/m6Am levels in these experiments (Figure S7F, G), presumably due to indirect effects, e.g., ZBTB48 overexpression increases FTO expression levels, as detailed in our manuscript (Figure S7C). Finally, as shown in Figure 3D, miCLIP-based quantification of m6A/m6Am levels in FTO and ZBTB48 overexpression cells also showed effects on global m6A/m6Am levels.*

*Regarding point 2: while RNA-MS and miCLIP experiments showed similar levels of m6A change for both FTO and ZBTB48 depletion (or overexpression), the signals obtained with these methods are presumably dominated by m6A sites that are co-regulated by these proteins. In fact, we found that ~50% of m6A containing genes show a lack of m6A sites in FTO and ZBTB48 overexpression cells, suggesting that ZBTB48 regulates a subset of FTO regulated genes (Figure S7B). Interestingly, a recent study by Yu et al., 2023 (PMID: 37257451; Mol Cell) identified RBM33 to be required for ALKBH5 mediated m6A demethylation. Although, PAR-CLIP identified only a subset of sites to be coregulated by RBM33 and ALKBH5, siRBM33 and siALKBH5 showed similar m6A levels*

*(Figure 3G and J in Yu et al., 2023). Thus, we believe that the observed similar m6A levels in siZBTB48 and siFTO cells are likely due to m6A signals being dominated by the coregulated sites.*

*To address the reviewer’s concern, we have discussed these two issues in the discussion section (see highlight on page 21).*

(2) Considering the significance of how FTO achieves target specificity, can the author anticipate the extent of applicability of the proposed model? Does the interaction between

ZBTB48 and FTO also exist in various human and mouse cell lines? If confirmed, this discovery would hold substantial value for the field. This interaction at least needs to be confirmed in HCT116 cells used for tumour growth model in this study.

*Authors’ response: We have performed co-IP experiments in HCT116 cells, as well as in U2OS cells. Consistently, we observed a robust interaction between ZBTB48 and FTO in these cell types (Figures S8B and Figure S11E).*

Minor concerns.

(3) The authors realised that knockdown of ZBTB48 does not change FTO levels, whereas overexpression of ZBTB48 leads to elevated FTO. It is unclear about the rationale behind overexpression studies?

*Authors’ response: We conducted experiments using both ZBTB48 KD and overexpression systems. To address the reviewer’s concern, we have added the rationale for using the overexpression system (see highlight on page 12; also shown below).*

“*We initially utilized ZBTB48- and FTO-overexpressing cells in our experiments for consistency, because elevated FTO levels have previously been shown to significantly decrease cellular m6A levels (32).*”

(4) Considering the multifunctional nature of ZBTB48, it's important to disentangle transcriptional and post-transcriptional roles of ZBTB48 to draw conclusions. I appreciate the analysis conducted in this manuscript. Is it possible to overexpress an DNA-binding mutant of ZBTB48 or RNA-binding mutant of ZBTB48 proteins?

*Authors’ response: ZBTB48 contains 11 C2H2-zinc finger domains, each of which can potentially contribute or not contribute to its RNA-binding and DNA-binding. Given that multiple C2H2 domains can simultaneously bind DNA and/or RNA, it has remained difficult to pinpoint the exact residues/domains for the interaction of ZBTB48 with nucleic acids. Moreover, it has been shown for certain C2H2-ZNFs (e.g., TFIIIA and YY1) that the same domains function to bind RNA and DNA and that RNA structure also plays a role in their binding (e.g., PMID: 14603324 and PMID: 27369384). Thus, in our opinion, precisely mapping the RNA-binding ZnFs in ZBTB48 warrants a separate study, which should thoroughly examine the structural aspects of ZBTB48 binding to DNA and RNA.*

(5) In Fig 2B, the inner panel does not match the metagene profile regarding the difference.

*Authors’ response: We have corrected this mistake and removed the inner panel, which was erroneously placed in this figure.*

(6) In Fig 5H, there is a lack of experiments for comparison, i.e. siFTO and o/e FTO.

*Authors’ response: We have performed the experiments suggested by the reviewer (updated Figures S10C and S10E; text highlight on page 16).*

(7) Page 3, "Genome-wide studies" should be "Transcriptome-wide studies".

*Authors’ response: We have made this change, as suggested.*

Strengths: The biochemistry and molecular biology experiments are comprehensive and well designed. The analysis is robust, and the conclusions generally align with the presented data.

Limitations: Cell line-specific and/or species-specific interaction?

Advance: filling a gap in our knowledge of how FTO achieves target specificity.

Audience: Basic research in the field of RNA modifications and RNA Biology. My expertise: Bioinformatics, gene regulation by RNA m6A modification

*Authors’ response: We thank the reviewer for providing valuable feedback to improve our work. We believe we have addressed all the concerns raised by this reviewer.*

Reviewer #2:

In the manuscript by Nabeel-Shah et al. the authors identify ZBTB48 as a novel interactor of

FTO. They show that ZBTB48 helps recruiting FTO to mRNA as well as the noncoding RNA Terra. Their results further suggest that this recruitment is required for FTO to demethylated m6A and m6Am RNA modification in target RNAs. This affects cellular rates of RNA turnover. Furthermore, the mechanism is involved in repressing colorectal cancer cell growth.

Overall, the authors present a new role of ZBTB48 in m6A and m6Am mediated RNA metabolism. They also suggest a nice model of ZBTB48 action, via the recruitment of the demethylase FTO. These findings will be of interest for the general RNA community and might be also relevant for cancer treatment. However, I have some concerns about the quality and analysis of the obtained data, especially the iCLIP and miCLIP experiments. The concerns are detailed below and should be addressed before publication.

*Authors’ response: We thank the reviewer for providing helpful feedback to improve our work. Below we provide our response to each point that the reviewer has raised.*

Major concerns:

1) Figure 1B) I think that the results shown in the autoradiograph are not very convincing and suggest that the purification of ZBTB48 is not very clean. The radioactive signal covers the lane from 50 kDa to 200 kDa. The ZBTB48 alone is running around 80 kDa. If the purification is specific most of the signal should be above 80 kDa. Maybe it helps to also use higher RNAse concentrations: Having very short RNA pieces will allow to evaluate specificity of the purification, since protein-RNA complexes will just a bit above the size of the protein.

This also applies to Figures S1E,F.

This concern could also apply to the generated iCLIP libraries and indicates that at least part of the obtained reads does not originate from ZBTB48 crosslinked RNA. The validation of protein-RNA interactions with RIP shown in Figure S2E supports the quality of the iCLIP data. Here some control RNAs should be analyzed to show that the RIP is not unspecifically enriching any RNA.

*Authors’ response: We have replaced the CLIP-autoradiography experiments in question.*

*As shown in the updated Figure 1B (also shown below for convenience), under high RNase I concentration, the radioactive signal is mostly reduced to a single band corresponding to the predicted molecular weight of ZBTB48. To further confirm that the radioactive signal emerges from ZBTB48 and* *is not due to co-purifying RBPs and/or in vitro RNA-binding, we conducted additional experiments. In these experiments, we mixed equal numbers of non-UV-crosslinked GFP-ZBTB48 cells and UV-crosslinked HEK293 cells, lysed them together, and then used them as a control (mixed control) in CLIP autoradiography. If the radioactive signal was due to interactions in vitro between ZBTB48 and other proteins and/or RNAs in the UV-crosslinked ZBTB48 lanes, we would expect a similar signal in the mixed control lane. As shown in the Figure S1E (also shown below), the radioactive RNA signal was specific to the UV-crosslinked GFP-ZBTB48 samples, and no appreciable signal was observed in the mixed controls (see highlighted text on pages 6 and 7). Thus, in combination with the RNA over-digestion assays shown in Figure 1C, these new data confirm the specificity of ZBTB48 RNA-binding.*

*It should also be noted that we conducted these experiments multiple times, and the autoradiographs shown in the initial submission were representative images. We have now shown both autoradiographs from the experiments that were used to excise the membranes for duplicate library preparations. As can be observed from this updated Figure S1G (also shown below), most of the radioactive signal runs immediately above the predicted ZBTB48 size, and that was the area that was excised.*

*Finally, as suggested by the reviewer, we have now included negative controls in the RIP-qPCR experiments shown in the updated Figure S2E (highlighted on page 8).*

Updated Figure S1G	IP: ZBTB48

1

1

5

8

0

6

5

5

0

3

0

1

8

5

+

U

V

R

N

a

s

e

I

+

+

+

I

P

I

N

P

3

2

[

P

]

+

-

3

2

[

P

]

R

N

A

A

u

t

o

r

a

d

i

o

g

r

a

p

h

M

a

r

k

e

r

M

a

r

k

e

r

9

3

7

0

5

3

4

1

3

0

1

3

0

U

V

R

N

a

s

e

I

+

+

1

:

2

5

0

1

:

2

5

0

I

P

:

Z

B

T

B

4

8

1

7

0

3

2

[

P

]

R

N

A

A

u

t

o

r

a

d

i

o

g

r

a

p

h

2) Regarding the co-occurrence of m6A sites as well as FTO and ZBTB48 binding sites shown in Figures 2B, C, F and G. The CLIP signal is a lot affected by crosslinking bias and read mappability. Therefore, to make these results more convincing it would be important to include additional iCLIP datasets (published other RBPs) for comparison.

*Authors’ response: We have now included the suggested analyses using the iCLIP-seq data for the classic RBPs PTBP1, U2AF1, and MKRN2, as well as for another C2H2-ZNF protein, SP1. These proteins did not show any significant enrichment around m6A sites (see Figure S5E, Text highlight on page 10).* *Moreover, to examine the specificity of FTO signal enrichment around ZBTB48 binding sites, we used SP1 iCLIP data as a control, and found that the SP1 signal was not significantly enriched around ZBTB48 sites (Figure S3D; Text highlight on page 9).*

3) Regarding Figure 2D and related analyses: I very much like this experiment and the results obtained here! I just wondered where the remaining reads go in the ZBTB48 knockdown. The introns? Maybe this becomes clearer in the meta profile representation used in Figure 1F.

For me the changes in the 5' UTR look most dramatic. Maybe this means that ZBTB48 is most important for recruitment of FTO to m6Am sites. Therefore, I think it would be good to differentiate the analyses in the remainder of the manuscript for m6A and m6Am sites. I first step would be to treat sites in the 5'UTR separate from the CDS and 3'UTR sites in the following analysis.

*Authors’ response: Although, the number of unique reads is comparable between siNT and siZBTB48 samples, we normalized mapped reads to the size-matched input (SMI), i.e., CLIP/SMI. Thus, the plot depicted in Figure 2D shows relative enrichment of FTO signal over the input across two conditions and as such does not represent the distribution or density of the original mapped reads. To address the reviewer’s concern, we plotted the FTO CLIP signal as crosslinks per million, and generated plots similar to the one shown in Figure 1F. Again, the most striking difference was observed in the 5’UTRs in siZBTB48 cells, even though the signal was not normalized to SMI in these analyses (Figures shown below). The FTO signal in the introns remained unchanged, whereas 3’UTRs showed slightly more crosslinks than other regions. Since these new analyses do not add much value to the current manuscript in our opinion, and since the processed mapped reads are provided with the manuscript (used for the plots below) as GEO submission, we have not included them in the revised version for the sake of consistency.*

*We also plotted FTO CLIP/SMI signal over the introns and consistently found that FTO binding was relatively reduced in the introns for siZBTB48 samples, although the difference between the two conditions (i.e., siNT and siZBTB48) was less striking than for the other regions that are shown in Figure 2D. We have shown this new analysis below and included it in the revised manuscript as Figure S4D.*

*Based on their binding profiles, we also initially hypothesized that ZBTB48 likely functions in FTO’s recruitment to m6Am sites. However, given our analysis of the FTO CLIP data in siZBTB48, we found that ZBTB48 depletion affects FTO’s targeting to both m6A and m6Am sites (Figure 2G). Consistently, analysis using m6A data also revealed that both m6A and m6Am sites were impacted in ZBTB48 overexpression cells (Figure 3D). Thus, while it remains possible that ZBTB48 might be more important for recruiting*

*FTO to m6Am sites (and m6A sites in the 5’UTR), our data indicates that ZBTB48, in fact, modulates FTO’s targeting to both internal m6A and TSS-associated m6Am sites. To address the reviewer’s concern, we have now conducted additional analysis using miCLIP data differentiating between m6A sites within different RNA regions, i.e., 5’UTR, CDS, and 3’UTRs. For these analyses, we used only DRACH motif containing sites and consistently found that FTO- or ZBTB48 overexpression significantly reduces m6A levels in the examined RNA regions. These new analyses are shown in Figure S6E of the revised manuscript (also shown below).*

4) I have some concerns about the analysis of the miCLIP data. In Figure 3A antibody crosslinking in all conditions appears similar. Yet in panel B there seems less signal for FTO and ZBTB48 overexpression in all areas. Have there been more reads generated for the GFP control? Where is the rest of the reads going? I think it would be required here to identify peaks that significantly change between the conditions. Then ask do those peaks coincide with DRACH and where are they located. Also, in the Genome browser pictures the signal is going down at all locations. Why is that? Usually, miCLIP generates a lot of background peaks. These should be unchanged.

*Authors’ response: The CLIP autoradiographs usually are considered as semiquantitative only, since various factors can affect the intensity of the observed radioactive signal (e.g., exposure time). The autoradiographs shown in Figure 3A were unfortunately overexposed and hence do not provide measurable quantitative information. Moreover, the autoradiographs in Figure 3A were generated using total RNA, which is dominated by non-protein-coding RNAs (e.g., tRNAs, microRNAs, ribosomal RNAs etc.), whereas Figure 3B shows the signal for only protein-coding genes. Therefore, the signal intensity observed in Figure 3A cannot be used to infer m6A levels in protein-coding transcripts. Given that FTO-overexpression reduces cellular m6A, and we found that ZBTB48 also reduces m6A, it was expected that the antibody would pulldown less m6A under these conditions. Thus, in comparison with the control cells (GFPonly), there are less uniquely mapping reads for FTO- and ZBTB48-overexpression cells. Nevertheless, our differential analysis (shown in Figure 3D) does account for any differences in the library size.*

*We agree with the reviewer that the anti-m6A antibody can indeed generate background reads. Therefore, we restricted our differential analyses (Figure 3D) to high-confidence*

*DRACH-motif containing m6A sites and, to address the reviewer’s point, we have now conducted additional analyses using these sites only. Specifically, in addition to those analyses described above in our response to point 3, we performed differential analysis of miCLIP data, and then, using DRACH-motif sites only, while distinguishing between various transcript features, i.e., 5’UTRs, CDS, and 3’UTRs, examined transcriptomic m6A levels. The global m6A levels were consistently reduced in FTO- and ZBTB48overexpression samples, in comparison with the control. These new analyses are shown in Figure S6G (also shown below).*

5) I am a bit confused by the author's interpretation of the results shown in Figure 5E. For me this plot shows that target transcripts are less downregulated and less upregulated than non-targets? Basically, they are less regulated overall. In this context I also think that the representation in of the mean in figure 5E and F is misleading. Data distribution should be visible as violin or boxplot.

*Authors’ response: We have replaced the figure panels in question by violin and box plots which, as the reviewer says, are easier to understand.*

Minor points:

Figure 1D) I think the legend in the panel is confusing and does not add information.

Especially since its in a different order than the categories on the y-Axis.

*Authors’ response: We have removed the legend panel in Figure 1D, since it does, indeed, not add information.*

Figure S2D) I wondered if the authors controlled for mappability of reads when picking random sites. How do the authors account for that in iCLIP there will be less reads in introns compared to exons?

*Authors’ response: Random sites were indeed controlled for mappability. Random sites were picked within the immediate vicinity (within 200 nts) of the actual sites.*

5E,F boxplots would be more suitable than barplots.xx

Authors’ response: We have replaced the bar plots with box plots.

Figure 2I: Please do not use "+ve"

*Authors’ response: We have replaced ‘+ve’ with ‘positive’ now.*

Figures with Microscopy pictures of cells have no scale bars or way too small.

*Authors’ response: Scale bars have now been added.*

The model that is shown in figure 7 is somehow misleading as it shows FTO binding only to ZBTB48 and not to the RNA.

*Authors’ response: Thank you for pointing out this oversight. We have replaced the figure in question. The model now shows that FTO binds to both ZBTB48 and RNA.*

Overall, the authors present a new role of ZBTB48 in m6A and m6Am mediated RNA metabolism. They also suggest a nice model of ZBTB48 action, via the recruitment of the demethylase FTO. These findings will be of interest for the general RNA community and might be also relevant for cancer treatment. However, I have some concerns about the quality and analysis of the obtained data, especially the iCLIP and miCLIP experiments. The concerns are detailed below and should be addressed before publication.

*Authors’ response: We thank the reviewer for providing critical feedback. We believe we have now addressed all the concerns raised by this reviewer.*

**Second round of review**

**Reviewer #1:**

Many thanks for the authors' efforts to explore the literature and conduct additional experiments as well as analyses to support their findings. Now that I have carefully and thoroughly read this revised manuscript, I am generally satisfied with the responses. I only have two minor issues as detailed below. (1) There is one minor question that the authors may have overlooked: "Can the authors anticipate the extent of applicability of the proposed model? Does the interaction between ZBTB48 and FTO also exist in various human and mouse cell lines?" This question arises because ZBTB48 was not identified as part of the FTO-interactome in a proximity labelling assay conducted on human 293T cells (PMID: 34634806). Could the authors discuss why this discrepancy might have occurred and provide some insights for the readers? (2) Regarding the newly inserted text on Page 21, could the authors please add relevant references to the corresponding sentence?

**Reviewer #2:**

The authors have addressed all my comments and concerns. Congratulations to the nice work!

**Authors Response**

**Point-by-point responses to the reviewers’ comments:**

*Response: As requested, we have now addressed the concerns raised by Reviewer 1 in the discussion section of this revised manuscript.*
